# Emotion as a cross-layer mechanism in filter bubbles: a social-psychological perspective

**DOI:** 10.3389/fpsyg.2025.1740709

**Published:** 2025-12-16

**Authors:** Shengyu He, Yang Fan

**Affiliations:** 1 School of Public Affairs, Zhejiang University, Hangzhou, China; 2 School of Marxism, Hangzhou Normal University, Hangzhou, China

**Keywords:** filter bubble, echo chamber, selective exposure, motivated reasoning, emotion, social identity, algorithmic amplification, political polarization

## Abstract

In increasingly personalized media environments, individuals encounter information that aligns with their existing beliefs, raising concerns about polarization, intergroup hostility, and the erosion of shared political reality. This review synthesizes recent research from a social-psychological perspective, arguing that filter bubbles are not produced by algorithms alone but emerge through the recursive interaction of motivated cognitive processing, identity-based social network structures, and algorithmic amplification of behavioral and emotional cues. We identify emotion as an underrecognized yet central mechanism that operates across these layers: emotional states such as anger, threat, and defiant self-worth guide information seeking, reinforce group affiliation, and shape algorithmic recommendation patterns, thereby intensifying filtering dynamics and contributing to attitude extremization. By conceptualizing filter bubbles as systems of cognitive coherence, identity protection, and affective regulation, we propose a dynamic multi-level explanatory model and outline implications for interventions, including reflective reasoning strategies, weak-tie exposure, and approaches that address the emotional and identity foundations of information selection.

## 1 Introduction

We live in a highly personalized information environment. Digital platforms analyze users' preferences, interaction histories, and social ties to curate individualized content streams. While this improves efficiency, it also reorganizes the conditions under which people encounter and interpret public issues. As individuals increasingly see a world that reflects their own beliefs, a key concern arises: *do we lose the capacity to understand dissent and share a common political reality?* These questions lie at the center of contemporary debates about polarization, intergroup hostility, and the erosion of shared public discourse (Arora et al., 2022).

Within this context, the concepts of *filter bubbles* and *echo chambers* have become central. Filter bubbles describe environments in which algorithmic curation immerses users in attitude-consistent information (Pariser, 2011), whereas echo chambers emphasize active selection, where individuals choose to interact primarily with like-minded others (Sunstein, 2017). Both phenomena originate from classic psychological mechanisms of selective exposure and confirmation bias (Festinger, 1957; Nickerson, 1998), reflecting the human tendency to avoid belief-threatening information and maintain a coherent sense of self.

Recent research, however, has redirected the discussion from a purely technological account to a *psychological—social—algorithmic interaction model* (Geschke et al., 2019). Users do not merely receive information; they shape their own information environments. When individuals search for political content, they frequently employ stance-laden keywords, generating “self-produced filter bubbles” even before algorithmic sorting occurs (Ekström et al., 2024). Algorithms then reinforce these tendencies by amplifying prior behaviors, while social networks consolidate attitude homogeneity. Importantly, emotional states such as anger and perceived threat motivate individuals to seek supportive viewpoints, deepening group boundaries and identity-based antagonisms (Wollebæk et al., 2019). Conversely, traits such as openness can help maintain broader informational horizons (Matz, 2021). Thus, the filter bubble is not merely an informational condition, but also a form of *self-regulation and affective management*.

This review advances a unified framework that conceptualizes the filter bubble as a self-reinforcing loop composed of confirmation bias, identity signaling, affective motivation, and algorithmic feedback. From a social-psychological perspective, we synthesize current research to examine how filter bubbles shape perceptions of political reality, foster attitude extremization, and intensify intergroup conflict. We further evaluate potential psychological intervention strategies, such as diversity prompts, reflective reasoning, and weak-tie exposure.

Four themes characterize the latest developments in this field: (a) filter bubbles involve *triple filtering* across psychological, social, and technological levels; (b) information selection is *emotionally and contextually dependent*; (c) individuals vary widely in their susceptibility to filter bubbles; and (d) algorithmic recommendation systems *amplify* rather than initiate these patterns. Building on these insights, the present review highlights emotion as an underrecognized but central mechanism that operates *across* filtering layers, shaping what individuals seek, attend to, and come to accept as legitimate political reality.

This review conceptualizes emotion as a cross-layer mechanism operating at the cognitive, social, and algorithmic levels, shaping both individual attention and collective meaning formation. This perspective enables a more integrated understanding of how affective processes connect micro-level cognition with macro-level information structures.

The literature reviewed in this article was identified through searches of PsycINFO, Web of Science, and Google Scholar using terms such as “filter bubble,” “echo chamber,” “selective exposure,” and “motivated reasoning.” Particular emphasis was placed on research published in leading social psychology journals over the past 3 years.

## 2 From concepts to mechanisms: an interactive psycho-social-technological system

Filter bubbles and echo chambers were initially used to describe information homogenization phenomena in digital media (Pariser, 2011; Sunstein, 2017). Early views considered the information environment as “passively given,” with users trapped in algorithm-constructed “cognitive cocoons.” As research deepened, however, scholars realized that filter bubbles are the product of interactions among algorithm design, users' psychological preferences, and patterns of social relations (Bruns, 2019). Consequently, the theoretical perspective has shifted from a view of technological determinism to a psychological—social—algorithmic interaction model.

### 2.1 Individual-level filtering: cognitive bias and motivated reasoning

The first layer of filter bubbles stems from cognitive biases in individuals' information processing. People tend to choose evidence that confirms existing beliefs and ignore or reinterpret contradictory information (Nickerson, 1998). This is not merely a cognitive strategy but also has profound emotional motivation: confirming beliefs reduces uncertainty and maintains a sense of self-coherence (Festinger, 1957).

Motivated reasoning theory posits that individuals process information not to pursue truth but to arrive at conclusions that feel psychologically right (Kunda, 1990). When political issues become bound up with morality or identity, motivated reasoning markedly intensifies, leading individuals to treat counter-attitudinal information as a threat and enter a defensive mode (Finkel et al., 2020). This processing can produce “backfire reinforcement,” making attitudes more extreme after encountering dissent.

Self-esteem and self-construal styles further modulate selective exposure. Individuals with low self-esteem rely more on congruent information to reduce uncertainty, while those with independent self-construals rely more on personal belief consistency. Those with interdependent self-construals are more influenced by in-group information. Under algorithmic systems, these psychological tendencies may directly affect platform recommendation patterns (Knobloch-Westerwick and Westerwick, 2023). Thus, at the individual level, filter bubbles are actively chosen because they provide psychological stability and safety.

### 2.2 Social-level filtering: group identity and affective resonance

The second layer captures social-level filtering, reflecting individuals' preference to affiliate with others who hold similar attitudes and identities. Digital platforms make social boundaries visible and traceable (Boyd and Ellison, 2007), which facilitates the formation of attitudinally homogeneous clusters. Political attitudes are reinforced through repeated interaction, shared language, and affective resonance within these groups (Barnidge, 2017). Even when platforms provide diverse content, if a user's core interpersonal ties are homogeneous, social feedback will stabilize and intensify shared viewpoints. Indeed, empirical studies show that on platforms such as Facebook, homogeneity in one's social network contributes more to filtering outcomes than algorithmic personalization alone (Bakshy et al., 2015).

Social identity theory helps explain this dynamic: political attitudes serve as group identity markers (Finkel et al., 2020). Information is evaluated not only on epistemic grounds (true/false) but relational ones (from “us" or “them"). This relational evaluation explains why misinformation can circulate stably within identity-aligned communities while external fact-checks often fail to penetrate out-group boundaries (Del Vicario et al., 2016).

### 2.3 Algorithmic-level filtering: preference amplification and feedback loops

The third layer concerns the algorithmic systems that structure information visibility. Algorithms do not generate preferences, but they magnify and accelerate existing ones. Designed to maximize engagement, they infer users' attitudinal leanings and recursively adjust content delivery: expressed preferences lead to more similar recommendations, which further constrain exposure to heterogeneous information (Flaxman et al., 2016). Thus, algorithms operate as *preference amplifiers*, not originators (Wu, 2017).

Algorithmic curation also shapes perception through relational cues. Content labeled as “liked” or “shared by similar users” is perceived as more credible (Knobloch-Westerwick and Westerwick, 2023). The persistent traceability of interaction histories encourages individuals to maintain consistent positions (Chadwick, 2017), contributing to the formation of an “affective public sphere” structured by identity-based emotional cues (Papacharissi, 2015).

Moreover, search behavior itself becomes a driver of filtering. Stance-laden queries produce increasingly polarized result sets, forming “search-driven filter bubbles” (Ekström et al., 2024). As incidental exposure declines, political out-groups become psychologically distant and morally devalued (Thorson, 2020).

Importantly, the relative weight of social vs. algorithmic filtering is *context-dependent rather than mutually exclusive*. In private messaging networks and close-tie groups, social homophily may dominate; in personalized recommendation feeds and high-volume attention environments, algorithmic amplification may exert greater influence. Thus, filter formation should be understood as a *parameterized interaction* in which social structure provides the filtering baseline and algorithms modulate its intensity and speed.

### 2.4 An integrative model: the filter bubble as a structured cognitive environment

To further clarify the theoretical mechanisms discussed above, Table 1 summarizes key representative studies across the individual, social, and algorithmic levels.

**Table 1:** Key literature and theoretical contributions to filter bubble mechanisms.

**References**	**Level**	**Core contribution**
Festinger (1957)	Individual	Proposed cognitive dissonance theory, explaining why individuals avoid belief-inconsistent information to maintain self-coherence.
Nickerson (1998)	Individual	Identified confirmation bias as a pervasive cognitive strategy shaping selective exposure and belief reinforcement.
Kunda (1990)	Individual	Developed motivated reasoning theory, highlighting emotional and identity-driven information processing.
Knobloch-Westerwick and Westerwick (2023)	Individual	Demonstrated that self-esteem and self-construal modulate selective exposure under algorithmic conditions.
Boyd and Ellison (2007)	Social	Showed how social networking platforms make social boundaries explicit, influencing patterns of information access.
Barnidge (2017)	Social	Found that homogeneous social interaction environments reinforce attitude certainty and affective alignment.
Bakshy et al. (2015)	Social	Demonstrated that social network homogeneity contributes more to filtering than algorithmic personalization.
Flaxman et al. (2016)	Algorithmic	Showed that recommender systems reduce incidental exposure to heterogeneous news content.
Papacharissi (2015)	Algorithmic/Social	Introduced the concept of the affective public sphere, framing political communication as emotionally encoded identity performance.
Geschke et al. (2019)	Integrative	Proposed the triple-filter model, integrating psychological preference, social structure, and algorithmic amplification.

Filter bubbles arise from the convergence of multiple interrelated mechanisms: (a) cognitive preferences, whereby individuals selectively attend to and interpret information that confirms existing beliefs; (b) social-relationship structures, in which homogeneous networks and identity-based group affiliations reinforce shared perspectives; and (c) algorithmic amplification mechanisms, through which digital platforms learn and intensify users' prior preferences by continuously personalizing and filtering the information they encounter.

The triple-filter model (Geschke et al., 2019) explains how these factors interact to produce self-reinforcing information environments. Psychological needs initiate filtering; social structures stabilize it; and algorithms amplify and accelerate it. The result is a structured cognitive environment in which individuals perceive their own beliefs as normative and opposing views as deviant (Finkel et al., 2020).

Therefore, filter bubbles cannot be dismantled through content exposure alone. Effective strategies must jointly address motivational regulation, relational restructuring, and system-level design.

## 3 Discussion

From a social-psychological perspective, this review reexamines the filter bubble phenomenon, emphasizing that its roots lie in individuals' cognitive biases, social identity needs, and emotion-regulation patterns, which are amplified by digital platform mechanisms. The evidence reviewed shows that filter bubbles are the product of dynamic interweaving among multiple mechanisms within a psychological—social—technological system.

### 3.1 Gap: emotion as a cross-layer filtering mechanism

Although the “triple filtering” framework has reached a high level of theoretical maturity, we contend that *emotion*, a core construct in social psychology, remains systematically underestimated in explanations of filter bubble formation. In much of the existing literature, emotion is treated as a secondary effect of cognitive bias—used to explain selective exposure or affective friction in opinion conflict. However, we argue that emotion is not a byproduct of information processing, but a primary psychological driver that propels attitude consolidation and political extremization.

Indeed, even research situated within sociological and algorithmic perspectives implicitly acknowledges the centrality of emotion. Studies demonstrate that anger and perceived threat in online political interaction significantly intensify polarization within filter bubbles (Wollebæk et al., 2019). As a proximity-oriented emotion, anger reduces deliberative scrutiny and encourages rapid defensive judgment. Similarly, Ekström et al. (2024) show that search behaviors are often grounded in emotional predispositions: individuals with strong political identities tend to use morally charged or hostile search terms, prompting search engines to return confirmatory content and reinforcing an iterative loop of “emotionally consistent retrieval → recommendation → re-retrieval.”

Moreover, the structure of content inside filter bubbles is emotionally coherent rather than informationally random. Fear, for instance, increases vigilance toward perceived threats, rendering out-groups more easily construed as dangerous or irreconcilable (Finkel et al., 2020). Sustained exposure to antagonistic narratives does not merely affirm threat cognitively; it consolidates threat emotionally, cultivating a *defensive worldview* in which extreme stances are experienced not as radical, but as justified, necessary, or even morally righteous. Thus, emotion does not simply arise in response to filtered information—it actively drives the filtering process, making the filter bubble a system for the production and circulation of specific emotional states.

Yet, research at the individual level continues to conceptualize emotion primarily as a regulator of cognitive processing (Knobloch-Westerwick and Westerwick, 2023). This motivated-reasoning paradigm positions cognition as primary and emotion as auxiliary. However, identity-political practices demonstrate that emotion can reconstruct cognitive and interpretive frameworks themselves. When feminist groups respond to practices of “slut-shaming" by strategically reclaiming the label (e.g., “So what if I'm a ‘slut'?"), emotion—specifically, defiant self-worth—serves as a resource for identity reformation, reshaping boundaries of meaning and group belonging. Similar dynamics underlie polarized repertoires such as whataboutism and counter-stigmatization. These examples show that emotion does not merely tint political reasoning; it reconstitutes what counts as reasonable, shared, or possible.

Therefore, we suggest that emotion constitutes a crucial theoretical gap in current filter bubble scholarship. This gap persists not because emotion is peripheral, but because it is so pervasive that it is normalized and conceptually flattened. Moreover, emotion does not merely intensify filtering at any one level; it operates across levels, shaping perceptual priors, group meaning-making, and algorithmic feedback loops simultaneously. Hence, emotion should not be assigned to a single stratum within the triple filtering framework, but rather understood as a *cross-layer dynamic*—and potentially conceptualized as a fourth filtering logic in its own right.

### 3.2 Toward dynamic and multi-level explanatory models

Building on the recognition of emotion as a cross-layer filtering mechanism, we propose an integrative and dynamic perspective: the formation of filter bubbles is not the outcome of any single layer, but a recursive system jointly shaped by (1) motivated cognitive processing, (2) interpersonal and identity-based aggregation, and (3) algorithmic amplification of emotionally charged preferences. The key mechanism is the *cyclical reinforcement* across these levels, where emotion and identity expectations guide information seeking, interpersonal alignment, and algorithmic feedback in mutually reinforcing ways.

For instance, even in the absence of algorithmic recommendation, users' stance-laden and affectively charged search terms can independently drive ideological bias (Ekström et al., 2024). This suggests that algorithmic diversification alone is insufficient for mitigating polarization, because the emotional and identity-based priors that guide search behavior remain intact. Likewise, informational interventions that attempt to “correct” attitudes without addressing the emotional and identity stakes involved can provoke defensive responses and backfire effects (Jenke, 2024).

Therefore, filter bubbles should be understood as *interaction systems* rather than as the linear effect of cognition, sociality, or algorithms alone. Emotion links and circulates across these layers, shaping what individuals seek, who they align with, and what platforms learn to amplify. This dynamic and recursive model underscores that efforts to reduce polarization must engage not only with informational structures, but also with the emotional and identity frameworks that organize how information becomes meaningful, acceptable, and shareable in the first place.

### 3.3 Limitations and future directions

While we have argued that emotion constitutes a crucial and currently underappreciated gap in filter bubble theory, it is also important to recognize that the broader literature—particularly that developed primarily in Anglophone research contexts—still contains several promising yet underdeveloped directions. These directions may lie somewhat outside the core social-psychological framing emphasized in this article, but their advancement has the potential to significantly enrich and extend affect-centered explanatory models.

First, existing empirical evidence still relies predominantly on cross-sectional observations and platform trace data, with limited longitudinal or experimental designs capable of establishing causal mechanisms (Rossini, 2023). Future work could integrate longitudinal psychological assessments with real-time behavioral data to examine the sequential dynamics of *attitude reinforcement*→*emotional response*→*information selection*, thereby directly tracing the recursive feedback loops theorized in this paper.

Second, recent scholarship suggests that different media ecologies do not contribute equally to the formation and intensification of filter bubbles. “Semi-private political discussion” environments such as WhatsApp or Telegram are often characterized by stronger relational intimacy, affective contagion, and in-group resonance (Vaccari and Valeriani, 2021), potentially producing more durable and emotionally charged polarization than public-facing platforms. This highlights the need to extend filter bubble research beyond public social media to include peer-to-peer and small-group networked communication (Chadwick et al., 2024).

Third, emerging research shows that users are not passive recipients of algorithmic outputs. Rather, they actively construct *folk theories of algorithms* and adapt their behaviors based on their assumptions about how platforms select and prioritize information (Toff and Nielsen, 2018). This suggests that future interventions should not focus solely on altering algorithmic recommendation patterns, but also on reshaping users' psychological models of algorithmic agency and influence.

Finally, recent advances in socio-affective intervention research highlight the importance of emotional awareness and meta-cognitive reflection as mechanisms to reduce defensive motivated reasoning and promote cross-group understanding. For example, affect-recognition and cognitive—affective framing interventions have shown promise in mitigating moral disengagement and improving intergroup dialogue in adolescents (D'Errico et al., 2024, 2025). Integrating such emotion-focused approaches with studies of algorithmic personalization may help future research develop more holistic frameworks for addressing the psychological roots of information selectivity. This direction is particularly important because it moves beyond treating users as passive targets of algorithmic influence, instead framing them as active emotional agents whose reflexive capacities can be cultivated to restore deliberative openness and epistemic resilience in digital environments.

Taken together, these directions point toward a research agenda that is methodologically dynamic, media-ecology sensitive, and attentive to the active role of users—and that complements, rather than replaces, the emotional and identity-centered framework we have advanced.

## 4 Conclusion

Filter bubbles are not merely the consequence of algorithmic personalization; they emerge from the dynamic interplay of cognitive biases, social identity needs, emotional regulation, and platform-level amplification. This review highlights that emotion is not a secondary or peripheral variable but a cross-layer force that shapes what individuals seek, how groups cohere, and what algorithms learn to reinforce. When filter bubbles provide psychological security and identity affirmation, they can stabilize meaning and belonging; yet, when they become emotionally charged and morally polarized, they intensify out-group hostility and erode the possibility of shared public reality. Therefore, addressing filter bubbles requires more than exposing individuals to opposing views or designing neutral recommendation systems. It calls for interventions that engage with the emotional and identity foundations of information processing, reshape social interaction contexts, and recalibrate algorithmic reinforcement loops. By conceptualizing emotion as a cross-layer mechanism in digital selectivity, this review bridges theoretical psychology with applied interventions for misinformation resilience and intergroup dialogue, suggesting that affect-centered frameworks can inform both cognitive training and design strategies in online communication. By centering emotion as a constitutive mechanism, future research can move toward models that better capture the lived, affective experience of political information environments—and toward strategies capable of sustaining dialogue across difference.
